# Training community health workers to screen for cardiovascular disease risk in the community: experiences from Cape Town, South Africa

**DOI:** 10.5830/CVJA-2016-077

**Published:** 2017

**Authors:** Thandi Puoane, Shafika Abrahams-Gessel, Thomas A Gaziano, Naomi Levitt

**Affiliations:** School of Public Health, Faculty of Community and Health Sciences, University of the Western Cape, South Africa; Center for Health Decision Science, Harvard TH Chan School of Public Health, Boston, MA, USA; Center for Health Decision Science, Harvard TH Chan School of Public Health, Boston, MA, USA ; Chronic Disease Initiative for Africa, Division of Endocrinology and Diabetes, Department of Medicine, University of Cape Town, South Africa; Chronic Disease Initiative for Africa, Division of Endocrinology and Diabetes, Department of Medicine, University of Cape Town, South Africa

**Keywords:** community health workers, non-communicable diseases, training, primary healthcare, cardiovascular diseases, screening

## Abstract

**Introduction:**

This article describes a training process to equip community health workers (CHWs) with knowledge and skills to identify individuals at high risk for cardiovascular disease (CVD) in a township in Cape Town.

**Methods:**

CHWs were employed by a non-governmental organisation (NGO) primarily focusing on non-communicable diseases (NCDs). They were trained in the theory of CVD, including physiological changes and related risk factors and in obtaining anthropometric and blood pressure measurements. Pre- and post-training tests assessed learning needs and the effectiveness of imparting knowledge about CVD, respectively.

**Results:**

Training increased knowledge about CVD risk factors. CHWs were able to screen and identify those at risk for CVD and refer them to health professionals for validation of scores. The initial one-week training was too short, given the amount of information covered. Some CHWs had difficulty with English as the primary instruction medium and as the only language in which tests were offered.

**Conclusion:**

Although CHWs could be trained to screen for CVD risk, increased training time was required to impart the knowledge. The language used during training and testing presented challenges for those trainees whose dominant, spoken language was not English.

## Introduction

Community Health Workers (CHWs) are individuals who live within the community they serve, understand the culture and speak the language of the people who live around them. In many low- and middle-income countries (LMIC), CHWs have been used largely to manage infectious diseases.

In South Africa, CHWs were initially used to promote better health in the communities during the 1980s. However, when the democratically elected government came to power in 1994, its plans to implement a national strategy for primary healthcare did not include using the services provided by CHWs, because it was claimed by the then minister of health that their services provided ‘second rate’ healthcare.^1^ As a result, the use of CHWs declined, although this was reversed by the government when the HIV epidemic placed increasing demands on the healthcare system. Now that South Africa is faced with a rising burden of non-communicable diseases (NCDs) in addition to injuries and HIV/AIDs, it is essential that the country expand its focus to include NCDs.

Indeed, in order to strengthen the current healthcare system, the South African government committed to re-engineering the primary healthcare system (PHC) as part of its strategic plan for NCDs from 2012 to 2016.^2^ It has undertaken to increase human resource capacity by using CHWs in the management of chronic conditions, with the goal of improving health outcomes.

The public health service provides care to about 80% of the population and focuses primarily on management and control of existing conditions and the prevention of complications among individuals with current diseases. There has been no active programme to provide early detection and management through screening for those at risk for NCDs. A large percentage of the population only visit a government health facility when they feel pain, at which point the disease has typically progressed.^3^

Based on the new public health approach,^4^ a more holistic, multidisciplinary and multi-sectorial approach is needed to improve the health of the population by tackling risk factors at the individual, community and societal levels. It is therefore important to build the capacity of services delivered by CHWs to include screening in the community, in order to identify those at risk before progression of the disease, and to provide appropriate referrals to health facilities for further assessment and appropriate treatment.

Over the past two decades, CHWs have been successfully used in HIV/AIDS programmes to provide palliative care to infected people. In addition, they have played an active role in the tuberculosis programme in areas such as directly observed treatment (DOT) and short-course medications for TB (tuberculosis-DOTs) to support adherence and retention in care.^5^

There is evidence that supports the role of CHWs in the control of already identified chronic disease.^6^ A recent review examined the effectiveness of CHWs in providing care for hypertension and noted improvements in keeping appointments, compliance with prescribed medication regimens, reduction in risk, blood pressure control and reduced related mortality rates.^7^

Studies also show that CHWs are effective in educating their communities about many health conditions, including CVD, diabetes and asthma.^8^,^9^ They can also help decrease healthcare costs, increase access to healthcare and information, and strengthen the family, community and local economy.^10^

Studies undertaken in China, Brazil, Iran and Bangladesh have shown that using CHWs can help improve health outcomes of the populations they serve.^11^ A study by the Health System Trust found that in communities with poor health services, the use of CHWs to conduct home visits led to an increase in the community’s access to relevant health information.^12^

Currently there is no standardised training curriculum for CHWs in South Africa. As a result, training is customised by individual organisations or institutions to equip CHWs to perform specific activities only over the lifetime of individual projects.^12^,^13^

This article describes the training process to equip CHWs with knowledge and skills to identify individuals who are at a high risk for CVD in Khayelitsha, a peri-urban township of Cape Town. The lessons learned and the challenges identified will inform the teaching methods/processes for training lay workers with varied educational levels. This will assist in the training of a knowledgeable workforce at the community level, which will play a role in addressing the escalating burden of NCDs. For the purpose of this article, a CHW is defined as a lay person who has received some training to deliver healthcare services but is not a health professional.

## Methods

The CHWs who participated in the training programme were recruited from an NGO that primarily focuses on delivery of services related to NCDs. It was envisaged that the knowledge acquired during the training would be utilised beyond the research period by CHWs in their daily activities. After the training, CHWs were expected to screen and identify people at risk for CVD in households in the community and refer them to four of the closest government health facilities to ensure that they received appropriate further treatment.

The study protocol was approved by the National Heart, Lung and Blood Institute (NHLBI), USA, as well as the respective institutional or ethics review boards in each of the four participating sites in the country. All staff members associated with the study successfully completed the ethics courses through the Collaborative Institutional Training Initiative (CITI).

In preparation for training, the trainers and the training coordinator visited the NGO to explain the purpose of the research and to recruit CHWs. The NGO agreed to release 10 to 15 CHWs from their daily activities to enable them to attend the training and participate in the field-work for the research project.

The actual selection of CHWs for the training was left to the coordinators at the NGO. They were asked to provide typical CHWs employed by them and not only the best performers. The NGO was financially compensated so that they could employ temporary replacements while the selected CHWs participated in training and field-work.

To break the ice and to get to know each other, the facilitators of the training briefly introduced themselves, followed by the trainees. In addition, each trainee presented his/her experiences in working with NCDs and expectations from the training. A summary of trainees’ expectations are listed in Table 1.

**Table 1:** Summary of community health workers’ expectations of the training

*Expectation*	*Rationale for expectation*
1. To acquire new skills related to chronic disease management	New skills would allow them to better serve their community’s NCD needs
2. To be provided with proper equipment	Proper equipment would improve their ability to assess the health of clients
3. To gain respect from the training	Completing the training at the university would mean that their community and peers would respect them more and regard them as legitimate health workers
4. To learn to provide assistance to persons recovering from chronic disease-related events	They needed to learn how to provide assistance to people who had suffered from stroke or complications from diabetes mellitus
5. To learn how to identify resources	They wanted to know how to identify government resources and assistance programmes that would allow them to assist the elderly and the young find food, safety and proper shelter
6. To learn how to function as part of a team	They wanted to learn how to work as part of a team that was responsible for providing care to the community, as they currently felt unsupported in this aspect of their work
7. To learn how to help sick clients who do not attend clinics despite their illness	They wanted to find ways to help their sick clients who were not able or willing to spend an entire day sitting at a clinic
8. To further their career goals	They aspired to becoming nurses, doctors or social workers and saw the completion of training as a stepping stone to these goals

The training was conducted for seven hours per week day from 1 to 12 June 2012. A total of 15 CHWs attended the training sessions. The training manual developed for the training included the teaching of theory about CVD and its risk factors, as well as the practical skills necessary to obtain blood pressure and other anthropometric measurements (weight, height). The training was held at the School of Public Health at the University of the Western Cape by three trainers who had content knowledge, experience in training and working with CHWs, fluency in the local language, could easily relate to the CHWs’ situation, and who had adapted the manual previously used to train the CHWs in various centres of excellence.

Based on the principles of andragogy, which uses life experiences and knowledge that adults bring to their learning experiences, the training was participatory in nature and designed to encourage sharing of experiences in delivering care for NCDs. Training materials were produced in English but isi-Xhosa was used extensively to provide culturally appropriate expansion of new, challenging concepts for the trainees.

**Fig. 1.**
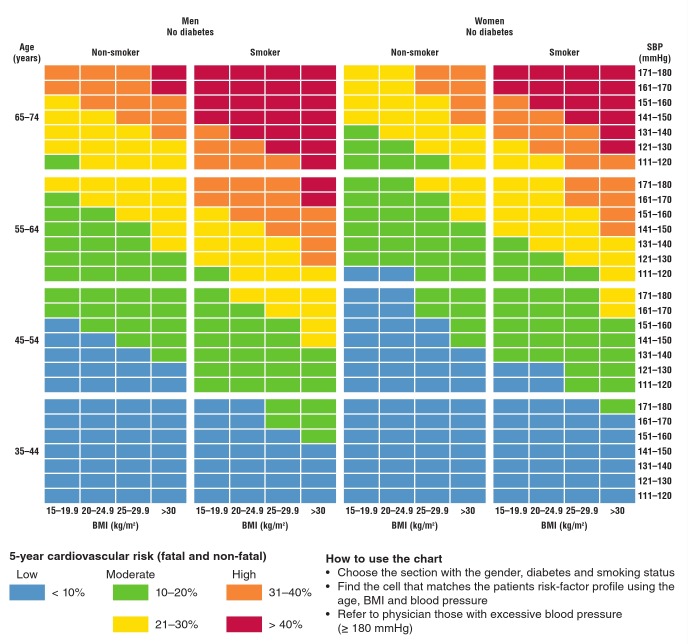
Risk-scoring chart used by community health workers to obtain CVD risk scores. Source: Gaziano TA, Young CR, Fitzmaurice G, Atwood S, Gaziano JM. Laboratory-based versus non-laboratory-based method for assessment of cardiovascular disease risk: the NHANES I follow-up study cohort. Lancet 2008; 371: 923-931.

A pre-training written test on the first morning of training was given to determine the current knowledge the trainees had on CVD and its risk factors and to assist in planning the teaching accordingly. During the first week, the morning sessions were spent in imparting knowledge about basic concepts of CVD and related risk factors. These included basic information about the heart and its function, hypertension, diabetes and obesity (definitions, symptoms and risk-factor history assessment) and nutrition using the South African food-based dietary guidelines. The afternoon sessions focused on data-collection skills, anthropometry measurements and training in the use of the non-invasive risk-screening tool (Fig. 1).

Trainees were taught to measure the mid-upper arm circumference (MUAC), used to determine the correct cuff size for measuring blood pressure using an automated Omron blood pressure machine®, as well as height and weight using an adjustable height stick and digital scale, respectively. Trainees were taught how to calibrate their blood pressure cuffs and digital scales. Calculation of body mass index (BMI) using a calculator, completion of risk-factor questionnaires through an in-person interview, maintaining confidentiality throughout the recruitment and screening process, assisting in the explanation and completion of informed consent forms, and completion of study forms for data entry were also included in the practical training.

At the end of the training, the trainees completed a posttraining written test, which was identical to the pre-training test. In addition they underwent a practical examination to assess their use of the risk tool to calculate CVD risk and to obtain anthropometric measures. For the former, the CHWs were given a scenario with all parameters needed to calculate a risk score (age, gender, smoking status, weight, height and average systolic blood pressure) and they had to calculate the CVD risk scores. The practical evaluations were completed individually by the study coordinator and trainers. Only CHWs who obtained 100% on both the use of the risk tool and obtaining anthropometric measurements and at least 60% on the content knowledge test were permitted to conduct screenings in the community.

A run-in period of two weeks prior to the start of the fieldwork allowed the study coordinator to assess the ability of CHWs who had qualified to perform screening. The coordinator randomly observed the CHWs during their screening activities and provided feedback at the end of the observations. During this two-week period, CHWs who were not able to perform adequately were withdrawn from further screening activities.

CHWs were given a third, post-field-work test six months after the training to assess longer-term knowledge retention. This test did not include evaluating the CHWs’ ability to obtain anthropometric measurements as they were required to hand in their equipment at the end of the field-work. All test scripts were graded, percentages of scores were calculated for each individual, and each participant was ranked. To maintain confidentiality, only numbers known to each individual were used for feedback.

## Results

A total of 15 CHWs (14 females) were recruited for the training, ranging between 23 and 36 years. All 15 trainees reported completing grade 12 but only three had passed the final examination for grade 12. All were receiving a stipend from the NGO (between R1 330 and R1 710 per month). The only previous training they had received was a two-day workshop related to providing support to clients in the management of their diagnosed chronic diseases but none related to screening for CVD.

**Fig. 2.**
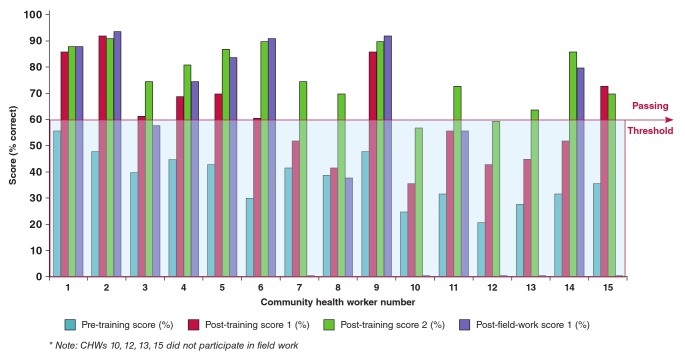
Pre- and post-test scores of community health workers.

The results of all written tests administered are shown in Fig. 2. Prior to the commencement of training, none of the 15 CHWs scored at least 60% (the passing grade) on the content knowledge test. Since the training was designed to be delivered over five working days, the first post-test was administered at the completion of the training programme (8 June 2012) in order to assess the effectiveness of the training in imparting knowledge. Only seven of the 15 trainees (47%) reached the passing threshold.

Based on these scores, it became clear that a second, additional test would be required in order for at least half of the trainees to be fit to conduct the field-work. A second week of training was added, focusing on areas identified by the training team as being poorly understood, through discussions with the trainees. A second post-training test was administered at the end of the second week (15 June 2012). This was identical to the first posttraining examination.

At the completion of the second week of training, 13 of the 15 trainees (87%) reached the passing threshold. During the two-week run-in period, two additional CHWs were removed from screening activities due to a failure to perform adequately in the community. Ten of the eleven CHWs continued fieldwork for the post-field-work testing. Out of these 10, 70% had maintained their knowledge levels above the passing threshold.

During field-work activities, the CHWs used a standard protocol^14^ to screen individuals aged 35 to 74 years for CVD risk and referred them to the health facility for further evaluation and treatment. Individuals with a measured systolic blood pressure > 180 mmHg were deemed clinically urgent cases and were given an urgent referral letter for immediate evaluation by a health professional at the closest health facility. They were ineligible for the CVD risk-assessment arm of the trial but were eligible for the referral arm.

A CVD risk score was calculated for all remaining participants using the risk-scoring tool (Fig. 1). Those whose risk score was > 20% were provided with non-urgent referral letters to see a health professional within two weeks of screening.

To validate the scores, a professional nurse who was a field coordinator calculated a second CVD risk score using de-identified data and was blinded to the CHWs’ risk scores and BMI calculations. Each CHW was expected to screen at least 100 people over a four- to six-week period at community screenings or in members’ homes. Table 2 provides information on the number screened and referred for treatment.

**Table 2:** Field-work activities of the community health workers

CHWs trained	15
CHWs selected for field-work	10
Community members screened	1 217
Community members with CVD risk > 20% (high risk)	7% of all screened persons
Persons provided with urgent referrals^1^	32.5% of all high-risk persons
Persons provided with non-urgent referrals^2^	67.5% of all high-risk persons

^1^Urgent referrals: screened persons advised to attend a health clinic on the day of screening.

^2^Non-urgent referrals: screened persons advised to attend a health clinic within two weeks of screening.

## Discussion

The process described in this article indicates that CHWs can be effectively trained in screening for CVD risk using a non-invasive screening tool. From the training process of the CHWs, three important points regarding the training were noted: (1) pre-training knowledge on NCDs; (2) language used during the training and in the written tests; and (3) knowledge retention over the longer term.

From the pre-training test in which no CHW scored more than 60%, it is clear that although they were working with NCDs, their knowledge of NCDs was limited. This raises concern about the appropriateness of current training received by CHWs in this area, given that the CHWs selected for this study were typical of those employed in the community by the largest NGO, so it is not clear what messages they were imparting to the community members during delivery of health services. It also raises questions as to who should be responsible for training the CHWs and how often they should be trained.

This is supported by a 2014 study by Tsolekile et al.,15 which found that in the absence of organised training for CHWs, most newly employed CHWs obtain their knowledge from their peers, who themselves do not always have or share correct information. In order for CHWs not to be viewed as providers of cheap or inferior-quality healthcare, they need to be properly trained. If properly trained they can provide an affordable first-contact level of care within the PHC system. Calculation of BMI was a new concept for the CHWs, even though obesity is a major risk factor for NCDs and a major public health problem in the South African population, especially among black women.^16^

Similarly, in 2011, Parker et al.,^17^ who examined knowledge about practical issues related to the prevention and control of non-communicable diseases, found that less than 10% of health professionals at primary-health facilities in the Western Cape attained a score of 80% and above. There is therefore a need for continuing training of health workers to keep them updated.

Although the CHWs were keen to be trained in English, it became clear that it was necessary to explain some of the difficult concepts in the local language commonly used by community members, including CHWs. This implies that training teams for CHWs should include trainers who are fluent in the local language of trainees, to ensure effective training. Furthermore, the burden of the requirement that all training materials must be produced and delivered in English is a potential barrier to effective training. Given that CHWs are trained to enable them to impart knowledge and skills to their communities using a local language, the training should be given in their local language.

A study that examined the challenges of facilitation in adult education found that facilitators are often not familiar with the language of the learners, which creates a barrier to proper learning, as some learners cannot check their understanding of the subject by communicating in their local language.^18^ Consideration should be given with regard to the purpose of using English to train this cadre of health professionals to accommodate trainers or learners.

Eighty-seven per cent of CHWs scored better in the second post-training test at the end of the second week of training than in the first post-training test administered at the end of the first week of training. This improvement is an indication that the challenges for CHWs in understanding new concepts were appropriately identified and addressed by the training team following exploration of the CHWs’ understanding of why the first week of training had failed.

The finding that 70% of the CHWs passed the post-field-work test demonstrates that effective training can facilitate long-term retention of new knowledge, and may also prove to be instructive when considering the need for retraining and the appropriate retraining intervals in future research. This is also an indication that people will remember activities that they perform on a daily basis. The decline in the percentage of CHWs who had retained the knowledge at the post-field-work test is similar to the findings of several studies that reported declining knowledge.^19^,^20^

From the training evaluation, it was clear that the training period was too short to cover all the new information. The CHWs reported that they experienced great difficulty in understanding the written examination tests in English rather than in their native isi-Xhosa, which was the language used to address conceptual challenges during the training itself. A retraining session including an extensive familiarisation of basic terminology in English correlating with the concepts under study was therefore necessary in order to get better scores. Further written tests should be administered in the CHWs’ first language or in English once proficiency has been demonstrated.

## Study limitations

While the ability to generalise the findings of this research is limited by its selection of CHWs working for a particular NGO located in an urban township in South Africa, these findings may not be different in other settings that employ CHWs and focus on NCDs due to lack of standardised training. Since the purpose of this training was to empower CHWs with skills to screen and identify those at risk for CVD, and refer them to the health facility for treatment, counselling on lifestyle modification was not included in this training.

## Conclusion

Although CHWs could be trained in screening for CVD risk using a non-invasive risk-screening tool, adequate training time was needed to enable them to grasp important new concepts. The language used in both teaching and testing this cadre of workers should be taken into consideration when measuring their performance. Inadequate or inappropriate training may result in health workers failing to do their work as expected. Sustaining the newly acquired knowledge requires refresher training.
